# Enhancement of furan aldehydes conversion in *Zymomonas mobilis* by elevating dehydrogenase activity and cofactor regeneration

**DOI:** 10.1186/s13068-017-0714-3

**Published:** 2017-01-31

**Authors:** Xia Wang, Qiuqiang Gao, Jie Bao

**Affiliations:** State Key Laboratory of Bioreactor Engineering, East China University of Science and Technology, 130 Meilong Road, Shanghai, 200237 China

**Keywords:** *Zymomonas mobilis*, Furfural, 5-Hydroxymethylfurfural (HMF), Reduction, Alcohol dehydrogenase, Transhydrogenase

## Abstract

**Background:**

Furfural and 5-hydroxymethylfurfural (HMF) are the two major furan aldehyde inhibitors generated from lignocellulose dilute acid pretreatment which significantly inhibit subsequent microbial cell growth and ethanol fermentation. *Zymomonas mobilis* is an important strain for cellulosic ethanol fermentation but can be severely inhibited by furfural and (or) HMF. Previous study showed that *Z. mobilis* contains its native oxidoreductases to catalyze the conversion of furfural and HMF, but the corresponding genes have not been identified.

**Results:**

This study identified a NADPH-dependent alcohol dehydrogenase gene ZMO1771 from *Z. mobilis* ZM4, which is responsible for the efficient reduction of furfural and HMF. Over-expression of ZMO1771 in *Z. mobilis* significantly increased the conversion rate to both furfural and HMF and resulted in an accelerated cell growth and improved ethanol productivity in corn stover hydrolysate. Further, the ethanol fermentation performance was enhanced again by co-expression of the transhydrogenase gene *udhA* with ZMO1771 by elevating the NADPH availability.

**Conclusions:**

A genetically modified *Z. mobilis* by co-expressing alcohol dehydrogenase gene ZMO1771 with transhydrogenase gene *udhA* showed enhanced conversion rate of furfural and HMF and accelerated ethanol fermentability from lignocellulosic hydrolysate. The results presented in this study provide an important method on constructing robust strains for efficient ethanol fermentation from lignocellulose feedstock.

**Graphical Abstract:**

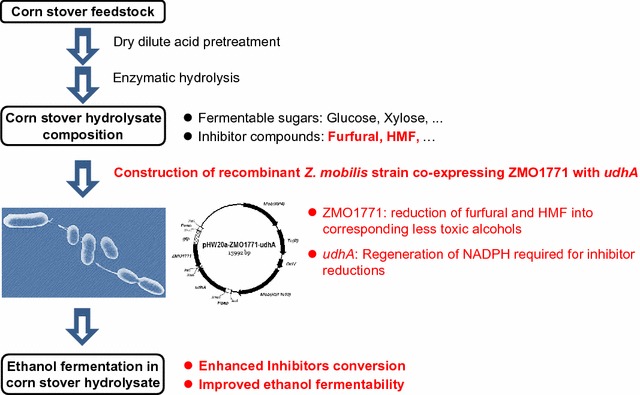

**Electronic supplementary material:**

The online version of this article (doi:10.1186/s13068-017-0714-3) contains supplementary material, which is available to authorized users.

## Background

Pretreatment is the crucial step to render lignocellulosic biomass to release fermentable sugars, but the harsh pretreatment conditions inevitably lead to the partial over-degradation of lignocellulose components and generate various small molecules with strong inhibition on subsequent microbial fermenting strains [1–3]. Among these inhibitor compounds, the two furan aldehydes, 2-furaldehyde (furfural) and 5-hydroxymethylfurfural (HMF) are the most toxic inhibitors for their strong toxicity to microorganisms and high contents in pretreated lignocellulose biomass [4, 5]. Fast and complete removal of furfural and HMF from pretreated lignocellulose (“detoxification”) by physical, chemical, or biological methods is strongly required to obtain the high conversion yield, titer, and productivity of ethanol product [1, 6, 7]. Ethanologenic strains with both high tolerance to furfural and HMF and high fermentability are an ideal consolidated solution for ethanol production [8].

Conversion of furfural (HMF) into less toxic furfuryl alcohol (HMF alcohol) by multiple NADH- and/or NADPH-dependent oxidoreductases is the primary detoxification pathway in microbial cells to eliminate the inhibition of furan aldehydes [9–11]. In *Saccharomyces cerevisiae*, over-expression of the oxidoreductase genes *ADH1*, *ADH6,* or *ARI1* improved the conversion of furfural or HMF and the ethanol productivity [12–15]. In *Escherichia coli*, furan aldehydes tolerance was enhanced by silencing of NADPH-dependent oxidoreductases (YqhD and DkgA) and over-expression of NADH-dependent FucO [9, 16].

*Zymomonas mobilis* is a natural ethanologenic facultative anaerobic strain and has many desirable industrial characteristics, such as higher specific rate of sugar uptake, high ethanol productivity, high ethanol tolerance, lower biomass production, non-requirement of controlled oxygen addition during fermentation, and regarded as safe status [17, 18]. Other than the native ethanol production, *Z. mobilis* has been engineered for sorbitol, gluconic acid, levan, 2,3-butanediol, isobutanol, and other chemicals production. *Z. mobilis* has served as an ideal platform for future biomass biorefinery [19, 20], but its weak tolerance to furfural and HMF is the major drawback when applied for ethanol fermentation using lignocellulose feedstock containing furfural and HMF generated from pretreatment [21, 22]. Several efforts have been tried to improve the inhibitor tolerance in *Z. mobilis*. Inactivation of a global regulator *hfq* (ZMO0347) decreased the resistance to furfural, HMF, acetate, and vanillin [23]. Mutations to the global transcription sigma factor (σ^70^) *rpoD* enhanced the tolerance to furfural stress [24]. Yang et al. confirmed that the tolerance of *Z. mobilis* to furfural was enhanced by over-expression of the histidine kinase encoding gene ZMO1162, or by disruption of the Sigma 54 modulation protein encoding gene ZMO0038 or 1-deoxy-D-xylulose-5-phosphate synthase encoding genes ZMO1598 and/or ZMO1234. Either knockout of the expression of gene ZMO0282, ZMO0283 or ZMO0285 or down-regulation of the expression of gene ZMO0282, ZMO0283 or ZMO0285 also enhanced the furfural resistance of *Z. mobilis* [25]. Alternatively, the strategy of adaptive laboratory evolution (ALE) was also used for development of a higher furfural-tolerant strain in *Z. mobilis*, which showed higher tolerance under 3 g/L furfural stress condition [26]. These studies offered the methods and gene sources for improving *Z. mobilis* tolerance to furfural and HMF, but further efforts are still needed to meet the requirement of practical lignocellulose biorefining with high inhibitor contents in the pretreated feedstock.

Previous study showed that *Z. mobilis* can reduce furfural or HMF into corresponding furfuryl alcohol or HMF alcohol [22], which suggested that *Z. mobilis* might also contain the native alcohol dehydrogenases (ADH) or aldo-keto reductases (AKR) to catalyze the reduction of furfural and HMF, but the corresponding genes have not been identified. In present study, the gene ZMO1771 encoding NADPH-dependent alcohol dehydrogenase was confirmed to be responsible for the efficient reduction of furfural and HMF in *Z. mobilis* ZM4. Over-expression of ZMO1771 in *Z. mobilis* improved the conversion of furfural and HMF, as well as ethanol fermentability in corn stover hydrolysate. The co-expressing *udhA* with ZMO1771 by elevating the conversion of NADH to NADPH further enhanced its conversion capacity of the two furan aldehydes in *Z. mobilis*. This study provided an important method for the construction of robust ethanologenic strains for efficient ethanol production from lignocellulose feedstock.

## Results and discussion

### Reduction evaluation of alcohol dehydrogenase and aldo-keto reductase gene expression

*Z. mobilis* ZM4 is able to convert furfural and HMF into less toxic furfuryl alcohol and HMF alcohol by its native alcohol dehydrogenases (ADH) or aldo-keto reductases (AKR) at the low concentration of furfural and HMF [22]. To enhance the reduction capacity and the conversion rate of high level of furfural and HMF, all the available alcohol dehydrogenase genes (*adh*) and aldo-keto reductase genes (*akr*) were screened from the genome of *Z. mobilis* ZM4 (GenBank: AE008692.2) as the candidates for over-expression in *Z. mobilis*. Totally twelve genes were identified, including seven *adh* genes (ZMO0062, ZMO1236, ZMO1596, ZMO1696, ZMO1722, ZMO1771 and ZMO1993) and five *akr* genes (ZMO0976, ZMO1344, ZMO1673, ZMO1773 and ZMO1984). Among these genes, ZMO1236 encoding ADHI and ZMO1596 encoding ADHII on the ethanol synthesis pathway already keep at high transcriptional levels during ethanol fermentation; thus, the two genes (ZMO1236 and ZMO1596) were excluded from the list. Rest of the ten genes were fused with the reporter gene *gfp* into pHW20a (Additional file 1: Figure S1) to yield ten *Z. mobilis* recombinants. The obtained recombinants harboring the expression cassette were confirmed by both fluorescence detection at 488 nm and enzyme activity assay (Table 1), and then cultured in RM medium containing 2 g/L of furfural or 4 g/L of HMF (Table 2).

**Table 1:** Gene expression detection by fluorescence and enzyme assay of cell-free extract in recombinant strains

	Fluorescent detection^a^	Activity (mU/mg crude protein)			
		Furfural + NADPH	HMF + NADPH	Furfural + NADH	HMF + NADH
ZM4(pHW20a-*gfp*) (Control)	+	21.15 ± 0.81	11.12 ± 0.45	110.87 ± 3.82	100.83 ± 2.73
ZM4(pHW20a-ZMO0062)	+	24.53 ± 0.99	14.82 ± 0.05	175.22 ± 9.91	135.51 ± 6.61
ZM4(pHW20a-ZMO1696)	+	29.40 ± 0.85	13.62 ± 0.25	134.00 ± 2.83	105.14 ± 1.62
ZM4(pHW20a-ZMO1722)	+	23.87 ± 0.87	12.24 ± 0.00	104.51 ± 2.69	99.40 ± 6.91
ZM4(pHW20a-ZMO1771)	+	32.31 ± 1.46	18.22 ± 0.45	102.97 ± 2.65	100.85 ± 3.89
ZM4(pHW20a-ZMO1993)	+	22.16 ± 0.00	11.62 ± 0.00	115.46 ± 0.00	101.64 ± 4.96
ZM4(pHW20a-ZMO0976)	+	62.24 ± 7.65	15.97 ± 0.38	133.27 ± 6.70	110.50 ± 3.19
ZM4(pHW20a-ZMO1344)	+	21.64 ± 0.00	12.34 ± 0.45	108.83 ± 2.80	94.19 ± 4.76
ZM4(pHW20a-ZMO1673)	+	22.31 ± 0.50	13.25 ± 0.30	116.88 ± 5.01	106.93 ± 2.39
ZM4(pHW20a-ZMO1773)	+	21.66 ± 0.49	11.89 ± 0.29	134.06 ± 4.86	107.71 ± 3.24
ZM4(pHW20a-ZMO1984)	+	20.46 ± 0.83	11.37 ± 0.46	99.31 ± 0.00	86.39 ± 2.84

Standard deviations were derived from at least two independent determinations

^a^+: fluorescence can be detected under fluorescence microscope; −: no fluorescence under fluorescence microscope

**Table 2:** Cell growth, glucose consumption, ethanol formation, and furan aldehydes conversion of recombinant *Z. mobilis* strains

Inhibitors	Recombinant strains	Cell growth (OD_600_)	Glucose consumption (g/L)	Ethanol production (g/L)	Inhibitor conversion (g/L)
Furfural	ZM4(pHW20a-*gfp*) (Control)	0.361 ± 0.003	4.81 ± 0.58	1.37 ± 0.02	1.13 ± 0.09
	ZM4(pHW20a-ZMO0062)	0.387 ± 0.003	4.13 ± 0.26	1.36 ± 0.04	1.04 ± 0.03
	ZM4(pHW20a-ZMO1696)	*0.404 ± 0.000*	*4.28 ± 0.19*	*1.39 ± 0.02*	*1.18 ± 0.03*
	ZM4(pHW20a-ZMO1722)	*0.393 ± 0.004*	*4.78 ± 0.22*	*1.56 ± 0.06*	*1.34 ± 0.06*
	ZM4(pHW20a-ZMO1771)	*0.378 ± 0.027*	*4.60 ± 0.26*	*1.49 ± 0.00*	*1.51 ± 0.02*
	ZM4(pHW20a-ZMO1993)	0.359 ± 0.009	4.87 ± 0.03	1.44 ± 0.00	1.02 ± 0.02
	ZM4(pHW20a-ZMO0976)	0.362 ± 0.007	4.09 ± 0.05	1.37 ± 0.06	0.99 ± 0.05
	ZM4(pHW20a-ZMO1344)	0.380 ± 0.002	4.56 ± 0.04	1.58 ± 0.03	1.01 ± 0.03
	ZM4(pHW20a-ZMO1673)	0.314 ± 0.008	3.71 ± 0.01	1.09 ± 0.20	0.93 ± 0.03
	ZM4(pHW20a-ZMO1773)	0.293 ± 0.002	3.19 ± 0.24	1.17 ± 0.14	0.93 ± 0.16
	ZM4(pHW20a-ZMO1984)	0.348 ± 0.009	4.80 ± 0.09	1.39 ± 0.09	1.00 ± 0.02
HMF	ZM4(pHW20a-*gfp*) (Control)	0.419 ± 0.005	6.47 ± 0.04	2.26 ± 0.14	1.40 ± 0.09
	ZM4(pHW20a-ZMO0062)	0.439 ± 0.009	5.57 ± 0.25	2.12 ± 0.17	1.43 ± 0.02
	ZM4(pHW20a-ZMO1696)	*0.497 ± 0.014*	*6.48 ± 0.07*	*2.04 ± 0.13*	*1.67 ± 0.08*
	ZM4(pHW20a-ZMO1722)	*0.427 ± 0.021*	*6.02 ± 0.04*	*1.89 ± 0.05*	*1.85 ± 0.03*
	ZM4(pHW20a-ZMO1771)	*0.471 ± 0.001*	*5.96 ± 0.24*	*2.41 ± 0.01*	*1.89 ± 0.04*
	ZM4(pHW20a-ZMO1993)	0.419 ± 0.001	5.92 ± 0.42	2.16 ± 0.08	1.19 ± 0.08
	ZM4(pHW20a-ZMO0976)	0.466 ± 0.005	6.95 ± 0.04	2.38 ± 0.05	1.41 ± 0.05
	ZM4(pHW20a-ZMO1344)	0.396 ± 0.020	5.96 ± 0.94	2.18 ± 0.04	1.65 ± 0.21
	ZM4(pHW20a-ZMO1673)	0.356 ± 0.013	5.44 ± 0.10	2.01 ± 0.04	1.16 ± 0.01
	ZM4(pHW20a-ZMO1773)	0.306 ± 0.009	5.27 ± 0.45	1.78 ± 0.04	1.03 ± 0.00
	ZM4(pHW20a-ZMO1984)	0.400 ± 0.002	5.20 ± 0.05	2.23 ± 0.03	1.55 ± 0.07

In RM medium containing 2 g/L of furfural or 4 g/L of HMF at 30 °C. ZM4(pHW20a-*gfp*) is the control strain only expressing the reporter gene *gfp*. The fermentation performance was detected at the mid-log phase, where 12 h for furfural and 16 h for HMF. Standard deviations were derived from at least two independent determinations

The results indicated that the expression of ZMO1771, ZMO1696, and ZMO1722 enhanced the furfural and HMF conversion, in which the expression of ZMO1771 encoding an iron-dependent alcohol dehydrogenase showed the most significant enhancement. 1.51 g/L of furfural or 1.89 g/L of HMF was converted at the mid-log phase of the fermentation after ZMO1771 was over-expressed, leading to the increase of furfural conversion by 33.63% and of HMF conversion by 35.00%, comparing to the control without ZMO1771 over-expression (Table 2). However, the cell growth, glucose consumption, and ethanol production of the three recombinants above did not show significant improvement in the synthetic medium with furfural or HMF addition. The rest of the seven genes, including two *adh* genes (ZMO0062 and ZMO1993) and five *akr* genes (ZMO0976, ZMO1344, ZMO1673, ZMO1773, and ZMO1984), did not show significant enhancement on furfural and HMF conversion. The in vitro enzymatic analysis in previous study [27] as well as in present study (Table 1) showed ZMO0976 encoding xylose reductase possessed NADPH-dependent furfural and HMF reduction activity, but no enhancement was observed in *Z. mobilis* ZM4 by expressing ZMO0976. The phenomenon suggests that the in vitro capacity of an enzyme may not go to the in vivo performance in *Z. mobilis*.

**Fig. 1:**
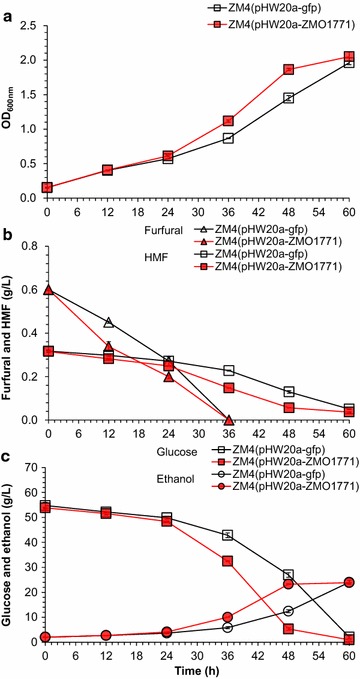
Fermentation performance of the recombinants *Z. mobilis* ZM4(pHW20a-ZMO1771) and ZM4(pHW20a-*gfp*) in corn stover hydrolysate. ZM4(pHW20a-ZMO1771), expressing gene ZMO1771 fused with the reporter gene *gfp*; ZM4(pHW20a-*gfp*), expressing the reporter gene *gfp* only. **a** Cell growth indicated by the OD value at 600 nm. **b** Conversion of furan inhibitors. **c** Glucose consumption and ethanol production. Conditions: 30 °C, static culture. The corn stover hydrolysate was prepared using the freshly dry dilute acid pretreated corn stover materials. The hydrolysate contained 56.74 g/L of glucose, 24.80 g/L of xylose, 2.85 g/L of acetic acid, 0.34 g/L of HMF, and 0.62 g/L of furfural. Mean values were presented with *error bars* representing at least two standard deviations

 Then the inhibitors conversion of all the ten recombinants was further evaluated in corn stover hydrolysate containing 56.74 g/L of glucose, 24.80 g/L of xylose, 0.62 g/L of furfural, 0.34 g/L of HMF, 2.85 g/L acetic acid as well as 4-hydromethylbenzoaldehyde, vanillin, syringaldehyde, and other inhibitors. The results again showed that the expression of ZMO1771 significantly increased furfural and HMF conversion rate (Table 3; Fig. 1): furfural by 0.26 g/L at 12 h and 0.40 g/L at 24 h, HMF by 0.03 g/L at 12 h, and 0.07 g/L at 24 h, respectively, while other gene expression did not show the obvious effect. The significantly difference between the corn stover hydrolysate and synthetic medium is that the cell growth, glucose consumption, and ethanol production increased by 35.00, 78.79, 100% at 36 h, and 33.33, 74.14, 100% at 48 h, respectively (Fig. 1). The results suggest that the expression of ZMO1771 significantly increased the furfural and HMF conversion as well as ethanol productivity in the high inhibitors containing corn stover hydrolysate.

**Table 3:** Furfural and HMF conversion rate of recombinant *Z. mobilis* strains in corn stover hydrolysate

Recombinant strains	Furfural conversion (g/L)		HMF conversion (g/L)	
	12 h	24 h	12 h	24 h
ZM4(pHW20a-*gfp*) (Control)	0.15 ± 0.01	0.33 ± 0.02	0.02 ± 0.01	0.05 ± 0.01
ZM4(pHW20a-ZMO0062)	0.15 ± 0.01	0.28 ± 0.02	0.01 ± 0.00	0.01 ± 0.00
ZM4(pHW20a-ZMO1696)	0.18 ± 0.00	0.33 ± 0.02	0.02 ± 0.00	0.05 ± 0.03
ZM4(pHW20a-ZMO1722)	0.18 ± 0.04	0.32 ± 0.05	0.02 ± 0.01	0.05 ± 0.01
ZM4(pHW20a-ZMO1771)	*0.26 ± 0.02*	*0.40 ± 0.01*	*0.03 ± 0.01*	*0.07 ± 0.00*
ZM4(pHW20a-ZMO1993)	0.16 ± 0.00	0.33 ± 0.01	0.01 ± 0.01	0.03 ± 0.00
ZM4(pHW20a-ZMO0976)	0.17 ± 0.02	0.31 ± 0.00	0.01 ± 0.00	0.03 ± 0.02
ZM4(pHW20a-ZMO1344)	0.14 ± 0.01	0.27 ± 0.01	0.01 ± 0.00	0.03 ± 0.01
ZM4(pHW20a-ZMO1673)	0.18 ± 0.00	0.29 ± 0.03	0.01 ± 0.00	0.06 ± 0.00
ZM4(pHW20a-ZMO1773)	0.13 ± 0.00	0.23 ± 0.01	0.02 ± 0.01	0.02 ± 0.01
ZM4(pHW20a-ZMO1984)	0.20 ± 0.02	0.31 ± 0.04	0.02 ± 0.00	0.04 ± 0.02

The corn stover hydrolysate contained 59.45 g/L of glucose, 24.03 g/L of xylose, 3.21 g/L of acetic acid, 0.38 g/L of HMF and 0.69 g/L of furfural at 30 °C. Standard deviations were derived from at least two independent determinations

Compared with the results in simple-defined RM medium, the improved conversion of furfural and HMF by expression of ZMO1771 may effectively alleviate the synergistic inhibition of furan aldehydes with other inhibitors in the hydrolysate, which then activate cell growth and ethanol production. Besides, the complicated hydrolysate components, such as the mineral element contents and other rich nutrients may also play a positive role to facilitate the reduction of furan aldehydes by ZMO1771, just as the previous studies on *S. cerevisiae* showed that enriched media, including complex media or high glucose concentrations, influence the inhibitor tolerance of cells [28, 29]. Thus, we recommend the use of a lignocellulosic hydrolysate for final fermentation evaluation of recombinant strains.

### Cofactor preference of ZMO1771 and reduction modification on furfural and HMF

The reductase enzymes for furan aldehydes conversion were either NADH or NADPH dependent, and the regeneration of the NADH or NADPH cofactor is an important factor for furan aldehydes conversion. The enzyme activity assay showed that furan aldehydes reductase activity of *Z. mobilis* ZM4(pHW20a-ZMO1771) increased by 52.8% on furfural and 63.8% on HMF when NADPH was used as the cofactor, while the activity was almost same to the control when NADH was used (Table 1). The cofactor preference of NADPH was further proved using the purified protein from the expression of ZMO1771 in *E. coli* BL21: the reductase activity was 3.632 U/mg protein on furfural substrate and 1.999 U/mg protein on HMF substrate when NADPH was used as the cofactor, while no reductase activity was detected when NADH was used. These results confirm that the ZMO1771 encoding enzyme is NADPH dependent for furfural and HMF reduction, rather than NADH.

Preference of NADPH by the relevant reductase enzymes on the furfural and HMF conversion pathway may negatively interfere with the NADPH/NADP^+^ balance of *Z. mobilis* and then weaken its tolerance to furfural and HMF. Additional NADPH supply may further enhance the furfural and HMF conversion of *Z. mobilis* when ZMO1771 is over-expressed. Two transhydrogenase genes *pntAB* and *udhA* from *E. coli* involved in NADPH generation were selected and co-expressed with ZMO1771 to facilitate the availability of NADPH. The two genes encode the only two transhydrogenases documented in living organisms responsible for the interconversion between NADH and NADPH (NADH + NADP^+^ ↔ NAD^+^ + NADPH) [30]. Each gene was inserted into pHW20a together with ZMO1771, but in reversed direction under the regulation of P*gap* promoter (Additional file 1: Figure S1). The PntAB and UdhA activities in the co-expression and the single expression of *pntAB* or *udhA* were essentially the same (0.037 U/mg protein for PntAB in both expressions, and 0.002 and 0.003 U/mg protein for UdhA activity, respectively), implying that each gene was expressed successfully in *Z. mobilis*.

The constructed recombinants were firstly evaluated in RM medium containing 2 g/L of furfural or 4 g/L of HMF (Table 4). 1.57 g/L of furfural or 2.72 g/L of HMF was converted by the co-expression of the transhydrogenase gene *udhA* with ZMO1771, leading to the increase of furfural conversion by 19.85% and of HMF conversion by 38.07%, respectively, over the cell with the expression of ZMO1771 only. The cell growth, glucose consumption, and ethanol production were improved significantly as well by 31.65, 21.93, and 35.68% under the tolerance to HMF, but only slight changes to furfural. However, the over-expression of the second transhydrogenase gene *pntAB* did not result in a significant change on furfural or HMF conversion in *Z. mobilis* (Table 4). The results suggest that *udhA* not *pntAB* favors the direction of NADPH production in the overexpression of ZMO1771 in *Z. mobili* where more NADPH might be required for furfural or HMF reduction, a similar function of *udhA* has been reported previously [31, 32].

**Table 4:** Cell growth, glucose consumption, ethanol formation, and furan aldehydes conversion of co-expression recombinant strains

Inhibitors	Recombinant strains	Cell growth (OD_600_)	Glucose consumption (g/L)	Ethanol production (g/L)	Inhibitor conversion (g/L)
Furfural	ZM4(pHW20a-ZMO1771)	0.259 ± 0.002	3.84 ± 0.11	1.14 ± 0.03	1.31 ± 0.25
	ZM4(pHW20a-ZMO1771-*pntAB*)	0.252 ± 0.011	3.44 ± 0.09	1.04 ± 0.02	1.49 ± 0.03
	ZM4(pHW20a-ZMO1771-*udhA*)	*0.243 ± 0.006*	*4.02 ± 0.59*	*0.91 ± 0.05*	*1.57 ± 0.27*
	ZM4(pHW20a-ZMO1771-ZMO0367)	0.230 ± 0.005	3.52 ± 0.34	1.14 ± 0.07	1.42 ± 0.11
HMF	ZM4(pHW20a-ZMO1771)	0.357 ± 0.004	6.43 ± 0.76	1.99 ± 0.08	1.97 ± 0.18
	ZM4(pHW20a-ZMO1771-*pntAB*)	0.421 ± 0.025	6.50 ± 0.13	2.28 ± 0.15	2.23 ± 0.14
	ZM4(pHW20a-ZMO1771-*udhA*)	*0.470 ± 0.036*	*7.84 ± 0.63*	*2.70 ± 0.08*	*2.72 ± 0.05*
	ZM4(pHW20a-ZMO1771-ZMO0367)	0.391 ± 0.009	5.66 ± 0.25	2.18 ± 0.16	2.02 ± 0.01

In RM medium containing 2 g/L of furfural or 4 g/L of HMF at 30 °C. The fermentation performance was detected at the mid-log phase, where 12 h for furfural and 16 h for HMF. Standard deviations were derived from at least two independent determinations

The co-expression of the gene ZMO0367 encoding glucose-6-phosphate dehydrogenase (G6PDH) with ZMO1771 in *Z. mobilis* was also evaluated. The G6PDH activity of the co-expression of ZMO0367 was considerably high (3.011 U/mg protein), but the co-expression of ZMO0367 with ZMO1771 did not show any enhancement on furfural and HMF conversion in RM medium comparing to the single expression of ZMO1771 (Table 4).

### Fermentation evaluation of the co-expression recombinants in corn stover hydrolysate

**Fig. 2:**
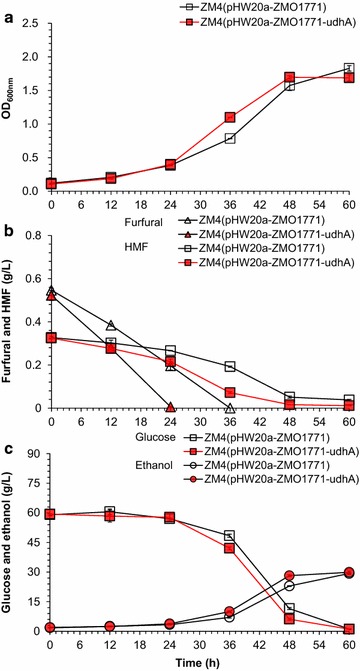
Fermentation performance of the recombinants *Z. mobilis* ZM4(pHW20a-ZMO1771-*udhA*) and ZM4(pHW20a-ZMO1771) in corn stover hydrolysate. ZM4(pHW20a-ZMO1771-*udhA*), co-expressing gene *udhA* with ZMO1771; ZM4(pHW20a-ZMO1771), expressing gene ZMO1771 only. **a** Cell growth indicated by the OD value at 600 nm. **b** Conversion of furan inhibitors. **c** Glucose consumption and ethanol production. Conditions: 30 °C, static culture. The corn stover hydrolysate was prepared using the freshly dry dilute acid pretreated corn stover materials. The hydrolysate contained 59.45 g/L of glucose, 24.03 g/L of xylose, 3.21 g/L of acetic acid, 0.38 g/L of HMF, and 0.69 g/L of furfural. Mean values were presented with *error bars* representing at least two standard deviations

The co-expression recombinant of *udhA* with ZMO1771 in *Z. mobilis* was grown in the corn stover hydrolysate containing 59.45 g/L of glucose, 24.03 g/L of xylose, 3.21 g/L of acetic acid, 0.69 g/L of furfural, and 0.38 g/L of HMF as well as other inhibitors (Fig. 2). The co-expression exhibited the maximum conversion capacity, approximately 50% of the initial furfural was reduced at 12 h and completely reduced at 24 h, 12 h shorter than the time used for complete conversion of furfural by the recombinant with the ZMO1771 expression only. The new recombinant also completely converted HMF within 48 h, comparing with 12% of the residual HMF left even after 60 h’s culture by the recombinant with ZMO1771 expression only. The cell growth, glucose consumption, and ethanol production were also accelerated by 55.56, 60.00, and 46.67% at 36 h, respectively; at 48 h, the accelerations were 10.00, 12.12, and 25.00%, respectively. The maximum cell growth and ethanol titer of the co-expression recombinant were approximately equal to the control at 60 h.

The Ethanol fermentation performance in corn stover hydrolysate revealed that the co-expression of *udhA* with ZMO1771 promoted the furfural and HMF reduction rate by elevating the NADPH availability by delivering the proton from NADH which was not the cofactor of ZMO1771 for furfural or HMF conversion reaction. This regeneration of NADPH from NADH by UdhA, however, could compete for NADH with the biosynthesis of ethanol from acetaldehyde by ADHI or ADHII and inhibit the ethanol formation rate when furfural or HMF conversion reactions proceed. Figure 2c showed this tendency of slow ethanol formation by *Z. mobilis* during the period of furfural and HMF conversion. When furfural was completely converted and most of HMF was converted, the recombinant *Z. mobilis* of the co-expression of ZMO1771 with *udhA* started its faster rate of ethanol generation than the wild *Z. mobilis* strain in which furfural and HMF existed at relatively high levels. The fermentation results of the co-expression of ZMO1771 with *udhA* suggest that cofactor manipulation was an effective tool for increasing the conversion of furan aldehyde inhibitors in *Z. mobilis*.

## Conclusions

The work in this study confirmed alcohol dehydrogenase ZMO1771 of *Z. mobilis* ZM4 possessed NADPH-dependent furfural and HMF reductase activity, which has never been reported before. Expression of ZMO1771 in *Z. mobilis* ZM4 significantly enhanced furfural and HMF conversion resulting in faster cell growth, glucose consumption, and ethanol production. In addition, the co-expression of another transhydrogenase gene *udhA* with ZMO1771 further increased its fermentation performance in hydrolysate by elevation of dehydrogenase activity and cofactor NADPH availability. Previous transcriptome study response to furfural revealed that the inhibitor tolerance of *Z. mobilis* involved multiple responses of cell membrane biogenesis, respiratory chain, DNA replication, DNA recombination and repair, transcriptional regulation, and some universal stress response [21]. The present work of identification of relevant oxidoreductase genes for furfural and HMF conversion is just a beginning. More genes involved in the tolerance to furan aldehyde inhibitors in *Z. mobilis* ZM4 should be identified, and then a higher tolerance strain could be developed by the combination of ZMO1771 with the new identified tolerance genes in the future. However, the robust strain with high furan aldehydes degradation constructed in this study would facilitate the efficient ethanol production from lignocellulose feedstock.

## Methods

### Strains, media, and culture conditions

The details of the strains and plasmids used in this study are given in Additional file 2: Table S1. *Z. mobilis* ZM4 (ATCC31821) and its recombinant strains were cultured at 30 °C without shaking in the rich medium (RM) containing 20 g/L of glucose, 2 g/L of KH_2_PO_4_, 10 g/L of yeast extract. 20 μg/mL of tetracycline was added to the RM medium where the recombinant strains were cultured. Pre-culture was prepared by inoculating one fresh colony in 5 mL of RM medium in 15 mL test tube and cultivated for 20 h to the stationary phase. All the culture was transferred into 50 mL of fresh RM in 250 mL flask and incubated overnight as the seed culture. 10% inoculum of the sub-culture was then adopted for all fermentation experiments.

*E. coli* strains were grown with Luria-Bertani (LB) medium at 37 °C. 50 μg/mL of kanamycin or 20 μg/mL of tetracycline was added to the medium accordingly. The shuttle vector pHW20a [33] was used for construction of *Z. mobilis* recombinants, and vector pET-28a(+) was used for expressing alcohol dehydrogenase ZMO1771 in *E. coli* BL21.

### Corn stover hydrolysate preparation

Corn stover was harvested from Dancheng, Henan, China in 2012. Cellulase enzyme Youtell#6 was purchased from Hunan Youtell Biochemical Co., Yueyang, Hunan, China. The filter paper activity, the cellobiase activity, and the protein concentration were 145 FPU/g, 344 IU/g, 90 mg/g enzyme, respectively [34].

Dry dilute acid pretreatment was used for corn stover pretreatment [35, 36]. Briefly, 2.5 g of sulfuric acid per 100 g of dry corn stover was co-currently fed into a 20 L pretreatment reactor with the ratio of the solid (the dry materials) to the liquid (the sulfuric acid solution) at 2:1 (w/w). The pretreatment was operated at 175 °C for 5 min under helically agitation at 50 rpm.

Corn stover hydrolysate was prepared in a 5 L bioreactor equipped with helical ribbon impeller for mixing. Freshly pretreated corn stover was hydrolyzed using cellulase at dosage of 15 FPU/g corn stover matter at 50 °C, pH 4.8 for 48 h. The slurry was centrifuged at 10,000 rpm for 10 min, and then the hydrolysate was autoclaved at 115 °C for 20 min and filtered by filter paper before use. No nutrients were added to the hydrolysate for all fermentation.

### DNA preparation, manipulation, and transformation

The genomic DNA of *Z. mobilis* ZM4 and *E. coli* K-12 were extracted using TIANamp Bacterial DNA Kit (Tiangen Biotech, Beijing, China). The oligonucleotides used for DNA amplification are listed in Additional file 3: Table S2. The constructed recombinant plasmids are given in Additional file 1: Figure S1. Each target oxidoreductase gene was amplified and fused with a reporter gene *gfp* via a short linker encoding seven glycines. The expression cassette was then inserted into vector pHW20a under the regulation of the P*eno* promoter. The constructed plasmid was introduced into *Z. mobilis* ZM4 using *E. coli* S17-1 λπ by biparental transconjugation [33].

ZMO1771 was also inserted in pET-28a(+) and expressed in *E. coli* BL21 for in vitro enzyme activity assay. The plasmid contained in frame *N*-terminal (His)_6_-tag before the start codon of the gene.

### Fermentation analysis of recombinant *Z. mobilis* strains

*Z. mobilis* recombinants were cultured both in 50 mL of RM medium or the freshly pretreated corn stover hydrolysate (without detoxification to remove the inhibitors contained) in 250 mL flasks by inoculating of 10% of seed culture as described above. 50% of the lethal furfural (2 g/L) or HMF (4 g/L) concentrations to the *Z. mobilis* cells were selected as the initial inhibitor concentrations in the RM medium before the fermentation started. Cell growth was determined periodically by measuring the optical density at 600 nm (OD_600_) using DU800 spectrophotometer (Beckman Coulter Inc., USA). The samples were withdrawn periodically and centrifuged at 12,000 rpm for 5 min and filtered through 0.22 μm filters. Glucose, ethanol, furfural, and HMF were analyzed using HPLC (LC-20 AD, refractive index detector RID-10A, Shimadzu, Kyoto, Japan) with an Aminex HPX-87H column (Bio-rad, Hercules, CA, USA) at 65 °C at the flow rate of 0.6 mL/min using 5 mM H_2_SO_4_ as the mobile phase.

### Protein purification and enzyme activity assays

Cells were collected at the exponential phase, and then the crude enzyme solution was prepared as described previously [37]. The his-tagged alcohol dehydrogenase encoded by ZMO1771 was induced by 0.1 mM IPTG and purified at 4 °C with Ni Aogarose 6FF resin (Aogma, Shanghai, China). Protein concentration was determined by Bradford protein assay using BSA as the calibration standard.

Furfural and HMF reduction activity was measured by monitoring the decrease in absorbance at 340 nm caused by NAD(P)H conversion at 30 °C [38]. One unit (U) was defined as the amount of enzyme converting 1 μmol of NAD(P)H per minute. Reaction mixture was performed in 100 mM phosphate buffer (pH 7.0) containing 100 μM NAD(P)H, 10 mM furfural or HMF. Glucose-6-phosphate dehydrogenase (G6PDH) and transhydrogenase (UdhA and PntAB) activity was determined at 30 °C by measuring the increase in absorbance at 340 nm and 375 nm, respectively. Reaction mixture for G6PDH contained 100 mM Tris-HCl (pH 7.5), 200 mM KCl, 10 mM MgCl_2_, 1 mM NAD(P), 2 mM glucose-6-phosphate [39]. Reaction mixture for UdhA and PntAB included 50 mM Tris-HCl (pH 7.6), 2 mM MgCl_2_, 0.5 mM NADPH, 0.5 mM 3-acetylpyridine adenine dinucleotide [30].

## Additional files

**Additional file 1: Figure S1.** Plasmids harboring functional genes used in this study. **a** pHW20a-*gfp*, the control plasmid. **b** plasmids harboring *adh* or *akr* genes. **c** plasmids harboring genes involved in NADPH generation.

**Additional file 2: Table S1.** Microbial strains and plasmids used in this study.

**Additional file 3: Table S2.** Oligonucleotide primers used in this study. The underlined letters indicated the restriction sites.

## Abbreviations

HMF: 5-hydroxymethylfurfural
HMF alcohol: 5-hydroxymethylfurfuryl alcohol
ADH: alcohol dehydrogenase
AKR: aldo-keto reductase
ARI: aldehyde reductase
ALDH: aldehyde dehydrogenase
G6PDH: glucose-6-phosphate dehydrogenase
ALE: adaptive laboratory evolution
IPTG: isopropyl β-d-1-thiogalactopyranoside
NADH: nicotinamide adenine dinucleotide (reduced)
NADPH: nicotinamide adenine dinucleotide phosphate (reduced)
NAD: nicotinamide-adenine dinucleotide
NADP: nicotinamide adenine dinucleotide phosphate
