# Supplementary material for: Enhancement of furan aldehydes conversion in Zymomonas mobilis by elevating dehydrogenase activity and cofactor regeneration
Source: Biotechnol Biofuels. 2017 Jan 31;10:24. doi: 10.1186/s13068-017-0714-3 (PMC5282692; doi:10.1186/s13068-017-0714-3)
Supplement: Supplementary file 2 — Additional file 2: Table S1. Microbial strains and plasmids used in this study. [file 13068_2017_714_MOESM2_ESM.docx]

**Additional file 2: Table S1 Microbial strains and plasmids used in this study.**

| **Strains and plasmids** | **Genotype, phenotype** | **Sources / references** |
| --- | --- | --- |
| **Strains** |  |  |
| *Z. mobilis* ZM4 | Wild-type strain, ATCC31821 | ATCC |
| *E. coli* K-12 substr. MG1655 | Wild-type strain, F^-^, λ^-^, ATCC 47076 | ATCC |
| *E. coli* BL21 (DE3) | F^-^ *ompT* *hsdSB* (*rB*^-^ *mB*^-^) *gal* *dcm* (DE3) | Novagen |
| *E. coli* S 17-1 λ π | *Pro*, *res*^-^, *mod*^+^; chromosomal integrated RP4, 2-*Tc*::Mu-*Km*::*Tn*7; *Tp*, *sm* | Simon et al., 1983 |
| **Plasmids** |  |  |
| pET-28a (+) | *Km^r^*, *ori* pMB1, T7 promoter/terminator | Novagen |
| pET-28a-ZMO1771 | Alcohol dehydrogenase gene ZMO1771 from ZM4 in pET-28a (+) | This study |
| pHW20a | *Tc*^r^, *mob* (RP4), *mob* (RSF1010), *lacZα*, MCS, and *ori*V | Dong et al., 2011 |
| pHW20a-P*eno* | Enolase (*eno*) promoter from ZM4 in pHW20a | This study |
| pHW20a-*gfp* | Reporter gene *gfp* in pHW20a-P*eno* | This study |
| pHW20a-ZMO0062 | Putative aryl-alcohol dehydrogenase gene ZMO0062 from ZM4 fused to *gfp* in pHW20a-P*eno* | This study |
| pHW20a-ZMO1696 | Zn-binding alcohol dehydrogenase gene ZMO1696 from ZM4 fused to *gfp* in pHW20a-P*eno* | This study |
| pHW20a-ZMO1722 | Zn-binding alcohol dehydrogenase gene ZMO1722 from ZM4 fused to *gfp* in pHW20a-P*eno* | This study |
| pHW20a-ZMO1771 | Alcohol dehydrogenase gene ZMO1771 from ZM4 fused to *gfp* in pHW20a-P*eno* | This study |
| pHW20a-ZMO1993 | Zn-binding alcohol dehydrogenase gene ZMO1993 from ZM4 fused to *gfp* in pHW20a-P*eno* | This study |
| pHW20a-ZMO0976 | Aldo-keto reductase gene ZMO0976 from ZM4 fused to *gfp* in pHW20a-P*eno* | This study |
| pHW20a-ZMO1344 | Aldo/keto reductase gene ZMO1344 from ZM4 fused to *gfp* in pHW20a-P*eno* | This study |
| pHW20a-ZMO1673 | Aldo-keto reductase gene ZMO1673 from ZM4 fused to *gfp* in pHW20a-P*eno* | This study |
| pHW20a-ZMO1773 | Aldo-keto reductase gene ZMO1773 from ZM4 fused to *gfp* in pHW20a-P*eno* | This study |
| pHW20a-ZMO1984 | Aldo-keto reductase gene ZMO1984 from ZM4 fused to *gfp* in pHW20a-P*eno* | This study |
| pHW20a-ZMO1771-P*gap* | glyceraldehyde-3-phosphate dehydrogenase (*gap*) promoter from ZM4 in pHW20a-ZMO1771 | This study |
| pHW20a-ZMO1771- *pntAB* | Proton-translocating pyridine nucleotide transhydrogenase gene *pntAB* from K-12 in pHW20a-ZMO1771-P*gap* | This study |
| pHW20a-ZMO1771-*udhA* | Soluble pyridine nucleotide transhydrogenase gene *udhA* from K-12 in pHW20a-ZMO1771-P*gap* | This study |
| pHW20a-ZMO1771-ZMO0367 | Glucose-6-phosphate dehydrogenase gene ZMO0367 from ZM4 in pHW20a-ZMO1771-P*gap* | This study |
| pHW20a-*pntAB* | *pntAB* from K-12 with *gap* promoter from ZM4 in pHW20a | This study |
| pHW20a-*udhA* | *udhA* from from K-12 with *gap* promoter from ZM4 in pHW20a | This study |
