# Supplementary material for: Enhancement of furan aldehydes conversion in Zymomonas mobilis by elevating dehydrogenase activity and cofactor regeneration
Source: Biotechnol Biofuels. 2017 Jan 31;10:24. doi: 10.1186/s13068-017-0714-3 (PMC5282692; doi:10.1186/s13068-017-0714-3)
Supplement: Supplementary file 3 — Additional file 3: Table S2. Oligonucleotide primers used in this study. The underlined letters indicated the restriction sites. [file 13068_2017_714_MOESM3_ESM.docx]

**Additional file 3: Table S2 Oligonucleotide primers used in this study.**

| **Primers** | **Forward and reverse sequences, respectively (5’-3’)** |
| --- | --- |
| P*eno*-F | ATAAGAATGCGGCCGCCTCGGCCATTGTCTATACTC |
| P*eno*-R | CCGCTCGAGATCGAAACCTTTCTTAAAATC |
| *gfp*-*Xho*I-F | CCGCTCGAGATGAGTAAAGGAGAAGAACTTTTCACTG |
| *gfp*- *Pst*I- R | AAAACTGCAGTTATTTGTATAGTTCATCCATGCCAT |
| *gfp*-R for ZMO0062 | ACGGGCGCTGTTTACCACCACCACCACCACCACCTTTGTATAGTTCATCCATGCCATGT |
| ZMO0062-F for *gfp* | GCATGGATGAACTATACAAAGGTGGTGGTGGTGGTGGTGGTAAACAGCGCCCGTTAGGA |
| ZMO0062-R | AAAACTGCAGTTAATCCGTTGGTAAAACGGC |
| *gfp*-R for ZMO1696 | AACCTATGGCGCGACCACCACCACCACCACCACCTTTGTATAGTTCATCCATGCCATGT |
| ZMO1696-F for *gfp* | TGGATGAACTATACAAAGGTGGTGGTGGTGGTGGTGGTCGCGCCATAGGTTATCAAAAG |
| ZMO1696-R | AAAACTGCAGTTAGAAGCCTTCTAAGACGATTTTACC |
| *gfp*-R for ZMO1722 | CAGCACGCGTTTTACCACCACCACCACCACCACCTTTGTATAGTTCATCCATGCCATGT |
| ZMO1722-F for *gfp* | CATGGATGAACTATACAAAGGTGGTGGTGGTGGTGGTGGTAAAACGCGTGCTGCTGTTG |
| ZMO1722-R | AAAACTGCAGTCAGAAAAGAACAACCGAACGA |
| *gfp*-R for ZMO1771 | AATCAAAATTGAGACCACCACCACCACCACCACCTTTGTATAGTTCATCCATGCCATGT |
| ZMO1771-F for *gfp* | AACTATACAAAGGTGGTGGTGGTGGTGGTGGTCTCAATTTTGATTATTATAATCCGACC |
| ZMO1771-R | AAAACTGCAGTTACAAGCTTGCAAGCAGAATAGC |
| *gfp*-R for ZMO1993 | CATAGGCTTCACTACCACCACCACCACCACCACCTTTGTATAGTTCATCCATGCCATGT |
| ZMO1993-F for *gfp* | GGATGAACTATACAAAGGTGGTGGTGGTGGTGGTGGTAGTGAAGCCTATGCGATTATCG |
| ZMO1993-R | AAAACTGCAGTTAAGGAATAAGTACCGTAGATCCG |
| *gfp*-R for ZMO0976 | GCGTAGAAGTGTTACCACCACCACCACCACCACCTTTGTATAGTTCATCCATGCCATGT |
| ZMO0976-F for *gfp* | GGATGAACTATACAAAGGTGGTGGTGGTGGTGGTGGTAACACTTCTACGCAAAAACCCG |
| ZMO0976-R | AAAACTGCAGTTATTTATCGCGTGGCGGG |
| *gfp*-R for ZMO1344 | TCGAAATAGGGGAACCACCACCACCACCACCACCTTTGTATAGTTCATCCATGCCATGT |
| ZMO1344-F for *gfp* | TGGATGAACTATACAAAGGTGGTGGTGGTGGTGGTGGTTCCCCTATTTCGATACCCTCT |
| ZMO1344-R | CGGGGTACCTTAAAATTCTTCATAGCGGGC |
| *gfp*-R for ZMO1673 | TTTTAACCGGCACACCACCACCACCACCACCACCTTTGTATAGTTCATCCATGCCATGT |
| ZMO1673-F for *gfp* | GGATGAACTATACAAAGGTGGTGGTGGTGGTGGTGGTGTGCCGGTTAAAAATGTAGTGC |
| ZMO1673-R | AAAACTGCAGTTAAAAATCGAAAACAGCCGG |
| *gfp*-R for ZMO1773 | GCTTCACATATTTACCACCACCACCACCACCACCTTTGTATAGTTCATCCATGCCATGT |
| ZMO1773-F for *gfp* | TGGATGAACTATACAAAGGTGGTGGTGGTGGTGGTGGTAAATATGTGAAGCTGCCGGAT |
| ZMO1773-R | AAAACTGCAGTTATAAGATAGCGAGATGCTGTTTTC |
| *gfp*-R for ZMO1984 | AATACGTATAATCACCACCACCACCACCACCACCTTTGTATAGTTCATCCATGCCATGT |
| ZMO1984-F for *gfp* | ATGAACTATACAAAGGTGGTGGTGGTGGTGGTGGTGATTATACGTATTTGGGTCGTACC |
| ZMO1984-R | AAAACTGCAGCTACCATGCATAGGCTTCAGG |
| P*gap*-F | CGAGCTCTTGGACTTTGTTCGATCAACAAC |
| P*gap*-R | TCCCCCCGGGATGTTTATTCTCCTAACTTATTAAGTAGCTAC |
| *pntAB*-F | TCCCCCCGGGGTACCATGCGAATTGGCATACCAAG |
| *pntAB*-R | CTAGTCTAGACATCCCGAATATTGGTTCAGTC |
| *udhA*-F | TCCCCCCGGGGTACCATGCCACATTCCTACGATTACG |
| *udhA*-R | CTAGTCTAGACTGCTGATGCTGGAAGATGG |
| ZMO0367-F | TCCCCCCGGGGTACCATGACAAATACCGTTTCGACG |
| ZMO0367-R | CTAGTCTAGAGCTAAACAGGGATTCTTCCATG |

The underlined letters indicated the restriction sites.
