# Supplementary material for: Enhancement of furan aldehydes conversion in Zymomonas mobilis by elevating dehydrogenase activity and cofactor regeneration
Source: Biotechnol Biofuels. 2017 Jan 31;10:24. doi: 10.1186/s13068-017-0714-3 (PMC5282692; doi:10.1186/s13068-017-0714-3)
Supplement: Supplementary file 3 [file 13068_2017_714_MOESM1_ESM.docx]

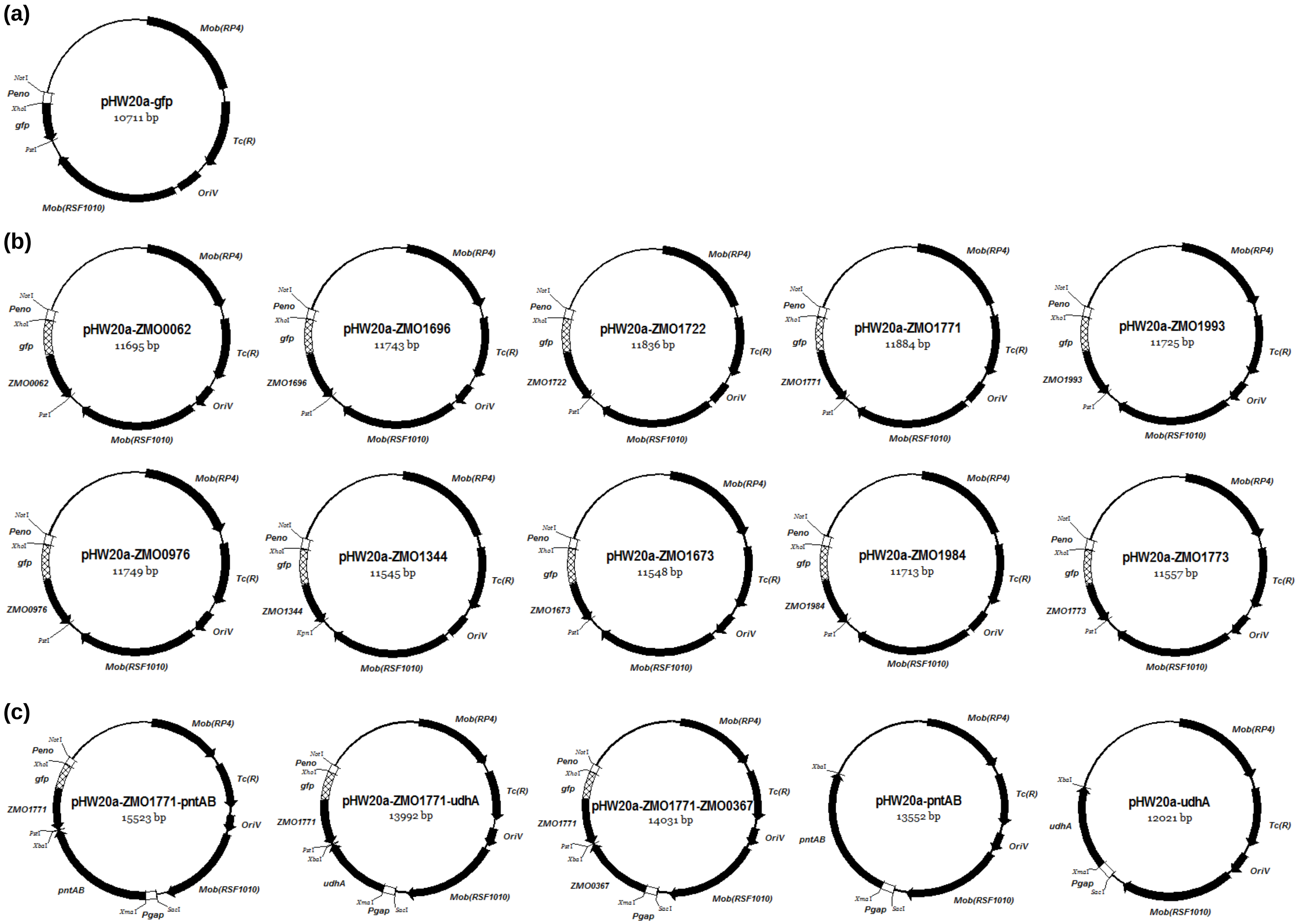
(a)
pHW20a-gfp
10711 bp
Mob(RP4)
Tc(R)
OriV
Mob(RSF1010)
NotI
Peno
XhoI
gfp
PstI
(b)
pHW20a-ZMO0062
11695 bp
pHW20a-ZMO1696
11743 bp
pHW20a-ZMO1722
11836 bp
pHW20a-ZMO1771
11884 bp
pHW20a-ZMO1993
11725 bp
pHW20a-ZMO0976
11749 bp
pHW20a-ZMO1344
11545 bp
pHW20a-ZMO1673
11548 bp
pHW20a-ZMO1984
11713 bp
pHW20a-ZMO1773
11557 bp
ZMO0062
ZMO1696
ZMO1722
ZMO1771
ZMO1993
ZMO0976
ZMO1344
KpnI
ZMO1673
ZMO1984
ZMO1773
(c)
pHW20a-ZMO1771-pntAB
15523 bp
pHW20a-ZMO1771-udhA
13992 bp
pHW20a-ZMO1771-ZMO0367
14031 bp
pHW20a-pntAB
13552 bp
pHW20a-udhA
12021 bp
XbaI
pntAB
udhA
ZMO0367
XmaI
Pgap
SacI
